# Locomotor muscle dysfunction and rehabilitative exercise training in fibrotic interstitial lung disease: Where are we at and where could we go?

**DOI:** 10.1113/EP091542

**Published:** 2025-10-08

**Authors:** Sarah Thivent, J. Alberto Neder, Anne‐Catherine Bernard, Marie Coudurier, Michel Guinot, Frédéric Hérengt, Samuel Verges, Mathieu Marillier

**Affiliations:** ^1^ Univ. Grenoble Alpes, Inserm, CHU Grenoble Alpes, Laboratoire HP2 Grenoble France; ^2^ Cardiopulmonary Rehabilitation Centre Dieulefit Santé Dieulefit France; ^3^ Laboratory of Clinical Exercise Physiology Queen's University and Kingston General Hospital Kingston Ontario Canada; ^4^ Unité Médicale Sports et Pathologies Service Hospitalo‐Universitaire de Pneumologie‐Physiologie, CHU Grenoble Alpes Grenoble France

**Keywords:** exercise training, muscle fatigue, muscle function, pulmonary fibrosis, pulmonary rehabilitation, skeletal muscle

## Abstract

Exercise limitation is a cardinal feature of fibrotic interstitial lung disease arising from pulmonary gas exchange, respiratory mechanical and cardio‐circulatory abnormalities. More recently, it has been recognized that impairment in locomotor muscle function (e.g., reduced muscle mass/strength or heightened fatigability) might also play a relevant contributory role. Exercise training as part of pulmonary rehabilitation is the most effective intervention to improve exercise tolerance, dyspnoea and quality of life in patients with fibrotic interstitial lung disease. Given that exercise training has modest effects on exertional ventilation, breathing pattern and respiratory muscle performance, improvement in locomotor muscle function is a key target for pulmonary rehabilitation in these patients. In the present narrative review, we initially discuss whether the locomotor muscles of patients might be exposed to negative risk factors. After offering corroboratory evidence on this matter (e.g., oxidative stress, inflammation, hypoxia, physical inactivity and medications), we outline their effects on skeletal muscle mass and functional properties. We finish by addressing the potentially beneficial effects of rehabilitative exercise training on these muscle‐centred outcomes, providing perspectives to facilitate or optimize the muscle benefits derived from this intervention. This narrative review, therefore, provides an up‐to‐date outline of the rationale for rehabilitative approaches focusing on the locomotor muscles in this patient population.

## Abstract (highlights)

- **What is the topic of this review?**
  This review focuses on risk factors for, recent evidence for, and relevance of locomotor muscle dysfunction in fibrotic interstitial lung disease. It also focuses on the ability of rehabilitative exercise training to counteract these pre‐specified abnormalities.
- **What advances does it highlight?**
  We highlight that fibrotic interstitial lung disease is characterized by multiple factors known to promote muscle dysfunction. We then discuss new evidence in favour of locomotor muscle dysfunction and its role in exercise limitation. Finally, we show that the exercise training offered to date minimal, inconsistent gains in muscle strength, emphasizing the need for alternative strategies to facilitate this intervention and combat existing impairments.

## 1 INTRODUCTION

Chronic fibrotic interstitial lung disease (*f*‐ILD) is a large group of restrictive ventilatory disorders encompassing >200 aetiologies characterized by a fibrotic destruction of the lung parenchyma. Idiopathic pulmonary fibrosis (IPF), the most devastating aetiology of *f*‐ILD, is thought to affect ∼3 million people worldwide, with a median survival of 3–5 years when untreated (Martinez et al., 2017). Exercise‐induced hypoxaemia, exertional dyspnoea and exercise intolerance are hallmarks of these diseases (Du Plessis et al., 2018; Smyth et al., 2023).

In this context, poor tolerance to physical exercise originates from impaired pulmonary gas exchange, abnormalities in respiratory mechanics and cardio‐circulatory abnormalities in this patient population (Agusti et al., 1991; Faisal et al., 2016; Hansen & Wasserman, 1996). However, in addition to the above‐mentioned impairments, recent evidence suggests that skeletal muscle dysfunction [such as reduced muscle mass and strength (Mendes et al., 2015) or increased fatigability (Marillier, Bernard, Verges et al., 2021b)] might play a significant role in exercise limitation in *f*‐ILD.

Exercise training, as part of pulmonary rehabilitation (PR), is arguably the most efficient strategy to improve functional exercise capacity, dyspnoea and the quality of life of patients with *f*‐ILD (Dowman, Hill et al., 2021). These beneficial effects (in comparison to no PR) are still evident 6–12 months after intervention. Consequently, participation in exercise‐based PR is strongly recommended as part of the non‐pharmaceutical management of patients with *f*‐ILD (Rochester et al., 2023). Beneficial changes in ‘respiratory responses’, such as peak‐exercise ventilation (Holland et al., 2008; Vainshelboim et al., 2014), breathing pattern (Vainshelboim et al., 2014) or static respiratory muscle strength (Zaki et al., 2023), have been reported following rehabilitative exercise training. However, such changes do not seem particularly large and are sometimes inconsistent; for instance, improvement in peak‐exercise ventilation averages 7 L/min (Dowman, Hill et al., 2021) and is not always reported (Dale et al., 2014), and the iso‐work rate ventilation does not seem to be reduced (Holland et al., 2008; Vainshelboim et al., 2014). It has been suggested that PR might ameliorate the above‐mentioned outcomes in *f*‐ILD by improving peripheral muscle function (Dowman, Hill et al., 2021), thus representing a key target for such intervention in these patients.

The present narrative review provides an up‐to‐date outline of the available literature supporting the relevance of skeletal muscle dysfunction and potential beneficial effects of rehabilitative exercise training to counteract these pre‐specified abnormalities in *f*‐ILD. To achieve these overarching goals, evidence is presented to answer three inter‐related questions:

1. What factors might contribute to locomotor muscle dysfunction in *f*‐ILD?
2. What is the current evidence for locomotor muscle dysfunction in *f*‐ILD?
3. Can rehabilitative exercise training reverse skeletal muscle abnormalities in *f*‐ILD?

We finish by presenting the rationale for different exercise training perspectives targeting the locomotor muscles, addressing a last question: how can the management of locomotor muscle dysfunction be optimized in *f*‐ILD? This endeavour might lead to original lines of research.

## 2 MATERIALS AND METHODS

### 2.1 Inclusion and exclusion criteria

The aim was to examine: (1) evidence for locomotor muscle dysfunction; (2) the effects of exercise training on muscle‐centred outcomes; and (3) the potential interests of alternative/adjunct training strategies in managing locomotor muscle dysfunction in *f*‐ILD. Studies were considered according to the following main criteria: (1) study of response to acute (a single bout) or structured and repetitive exercise (exercise training); (2) reporting at least one muscle‐centred outcome (i.e., mass, strength, endurance or fatigability); and (3) regardless of testing (e.g. isolated muscle vs. whole‐body exercise) or assessment (e.g., voluntary vs. evoked muscle strength) methods. The primary focus of this review is idiopathic interstitial pneumonias; evidence from secondary ILD (e.g., advanced fibrotic sarcoidosis) are also presented when deemed relevant. Additional studies are presented when they provide relevant context to this narrative review (e.g., if referring to mechanisms of exercise intolerance in *f*‐ILD). Selected studies needed to include original data and be published in English.

### 2.2 Search method

A screening of the literature, performed by two reviewers (S.T. and M.M.), examining locomotor muscle function and the influence of exercise training on muscle‐centred outcomes in *f*‐ILD was conducted up to July 2024 using the PubMed/MEDLINE database. A second screening was conducted in May 2025, coinciding with the revised version of the present narrative review. Our search strategy included: population of interest (interstitial lung disease OR idiopathic interstitial pneumonias OR idiopathic pulmonary fibrosis) AND intervention (exercise OR exercise training OR pulmonary rehabilitation) AND outcomes (muscle function OR muscle mass OR muscle strength OR muscle endurance OR muscle fatigue). This search strategy was complemented by a manual search of potential additional references together with authors’ knowledge of the literature.

### 2.3 Glossary

This section is intended to provide the non‐expert reader with some definitions of key concepts addressed in this narrative review. Muscle atrophy is defined as a decrease in muscle mass occurring when protein degradation exceeds synthesis (Fanzani et al., 2012), whereas sarcopenia refers to a progressive and generalized loss of muscle mass and strength in line with ageing (Cruz‐Jentoft et al., 2019). Muscle disuse is the primary process leading to muscle atrophy owing to decreased muscle contractile activity (Nunes et al., 2022). Malnutrition refers to deficiencies (or excesses) in nutrient intake, imbalance of nutrients or impaired nutrient utilization and is typically evidenced by a low body mass index or fat‐free mass index in *f*‐ILD (Jouneau et al., 2019). Muscle weakness refers to the loss of muscle strength [i.e., the ability of muscles to generate force (Gea et al., 2012)]. Muscle endurance broadly relates to the ability to maintain/repeat submaximal contractions over time, whereas muscle fatigue refers to any decrease in the ability to apply muscular force (or power) caused by exercise (Bigland‐Ritchie & Woods, 1984). The term fatigability can be used to normalize the level of muscle fatigue experienced by a subject relative to a given task (Gruet, 2018).

## 3 WHAT FACTORS MIGHT CONTRIBUTE TO LOCOMOTOR MUSCLE DYSFUNCTION IN FIBROTIC INTERSTITIAL LUNG DISEASE?

### 3.1 Ageing

Except forspecific aetiologies (e.g., sarcoidosis), most patients with *f*‐ILD are older adults (Patterson et al., 2017). For instance, the prevalence of IPF increases drastically after 65 years of age (Raghu et al., 2006). It is now well recognized that ageing plays a sizeable role in the deterioration of skeletal muscle mass and function. Indeed, sarcopenia has been largely described as a consequence of loss of muscle fibres, atrophy and reduced reinnervation capacity (in fast‐twitch fibres in particular), lower protein content and enzyme activity, changes in neural drive to the muscles, and mitochondrial loss and dysfunction (Cohen et al., 2015; Kostka et al., 2000; Proctor et al., 1998). Such muscle loss seems inevitable (even for exercise‐trained people) and accelerates after 50 years of age (Proctor et al., 1998), with a 25%–30% reduction in muscle mass in the seventh decade of life (Maltais et al., 2014).

The decrease in circulating levels of several anabolic hormones [e.g., growth hormone, testosterone, insulin‐like growth factor (IGF)‐I or dehydroepiandrosterone (DHEA) and its sulphate ester (DHEA‐S)] with ageing might also contribute to the abovementioned muscle loss (Guadalupe‐Grau et al., 2017; Kostka et al., 2000; Proctor et al., 1998; Valenti et al., 2004). This phenomenon might be particularly relevant in *f*‐ILD, because lower levels of IGF‐1 and DHEA‐S have been reported in patients versus age‐matched control subjects (Bloor et al., 2001; Mendoza‐Milla et al., 2013).

### 3.2 Oxidative stress

A sizeable body of evidence suggests that oxidative stress plays a key role in the pathogenesis of *f*‐ILD, contributing to lung tissue injury and fibrosis (Kinnula et al., 2005; Otoupalova et al., 2020) and correlating with disease severity (Daniil et al., 2008; Matsuzawa et al., 2015). Studies have reported higher levels of reactive oxygen and nitrogen species, leading to lipid peroxidation (Malli et al., 2013; Montuschi et al., 1998; Rahman et al., 1999), protein carbonylation (Rottoli et al., 2005) and enhanced nitrotyrosine [a by‐product of protein nitration (Saleh et al., 1997)] production in the lungs of patients versus healthy control subjects. Patients have also been found to have a lower antioxidant capacity in the plasma and bronchoalveolar lavage fluid, suggesting that the natural oxidant–antioxidant balance is disrupted in *f*‐ILD (Rahman et al., 1999), leading to the accumulation of reactive oxygen and nitrogen species at both the lung and systemic levels (Kinnula et al., 2005; Rahman et al., 1999).

Greater chronic accumulation of reactive oxygen and nitrogen species can also be detrimental at the skeletal muscle level (Scicchitano et al., 2018). Indeed, oxidative stress‐induced damage to lipids, proteins and DNA (Sies et al., 2017) can trigger several abnormalities, including impaired muscle fibre integrity and function (Andrade et al., 1998; Barbieri & Sestili, 2012; Jackson & O'Farrell, 1993), muscle protein breakdown and degradation (Aucello et al., 2009; Moylan & Reid, 2007), mitochondrial dysfunction (Barbieri & Sestili, 2012) and enhanced autophagic–apoptotic pathways in muscle cells (Aucello et al., 2009; Barbieri & Sestili, 2012). This can, in turn, impair muscle function and prompt muscle atrophy. Oxidative stress can also impair the function of satellite cells (muscle regeneration and repair) by reducing their proliferation and differentiation capacities (Gonzalez et al., 2021; L'Honore et al., 2018; Renault et al., 2002). We are not aware of studies specifically exploring the link between oxidative stress and muscle damage in *f*‐ILD.

### 3.3 Inflammation

Chronic lung inflammation is a hallmark of *f*‐ILD and is associated with a chronic systemic inflammatory response reflected by increased circulatory levels of inflammatory mediators, such as cytokines (Coker & Laurent, 1998; Hiraiwa, 2014). Experimental animal studies (Goodman, 1991, 1994; Shieh et al., 2019) and investigations in older adults (Schaap et al., 2006; Visser et al., 2002) have shown that these pro‐inflammatory cytokines can have a detrimental impact on skeletal muscle mass and strength. For instance, Shieh et al. (2019) found drastic quadriceps muscle atrophy in a mouse model of lung fibrosis, which might be attributable to the high circulating levels of interleukin‐6 (IL‐6) and interleukin‐33 (IL‐33) secreted from the injured lung tissue. Specifically, IL‐6 and IL‐33 activated signal transducer and activator of transcription‐3 and AMP‐activated protein kinase signalling, respectively, inducing the expression of muscle‐specific proteolysis markers [muscle RING‐finger protein‐1 and Atrogin‐1 (Shieh et al., 2019)]. In the elderly, higher concentrations of IL‐6 and tumor necrosis factor (TNF)‐α have been linked cross‐sectionally to lower muscle mass and strength (Visser et al., 2002), with elevated IL‐6 being associated with a 3‐fold increased risk of dynamic strength loss [>40% over 3 years (Schaap et al., 2006)]. On the contrary, TNF‐α could decrease muscle expression of IGF‐I and myogenic differentiation factor, thereby reducing skeletal muscle regeneration and inhibiting myogenic differentiation (Fernandez‐Celemin et al., 2002; Langen et al., 2004). Inflammation might thus contribute to skeletal muscle dysfunction by affecting the muscle protein synthesis–breakdown balance in *f*‐ILD.

### 3.4 Hypoxia

Early and severe exercise related‐hypoxaemia is a hallmark of *f*‐ILD (Du Plessis et al., 2018); >50% of patients may experience O_2_ saturation ≤88% during walking (Khor et al., 2019). Severe exercise‐related hypoxaemia worsens skeletal muscle susceptibility to fatigue in *f*‐ILD through an impairment in muscle O_2_ delivery (Marillier et al., 2025). In fact, muscles are exquisitely sensitive to any alteration in O_2_ delivery, the net result of which is a disturbance in muscle metabolism (Adams & Welch, 1980). Metabolites implicated in the development of skeletal muscle fatigue [e.g., H^+^ or inorganic phosphate (Allen et al., 1995)] build up faster when muscle O_2_ supply is reduced (Hogan et al., 1999), eventually causing an early impairment in muscle contractility (Amann & Calbet, 2008; Marillier et al., 2021). Of note, a faster rate of skeletal muscle deoxygenation is strongly correlated with lower exercise tolerance and greater leg muscle discomfort in patients with *f*‐ILD (Marillier, Bernard et al., 2023).

A lower fraction of patients with advanced *f*‐ILD show resting hypoxaemia (Khor et al., 2021). Both chronic hypoxaemia and tissue hypoxia have been associated with the extent of systemic inflammation, thus probably promoting muscle wasting (Baldi et al., 2008; Pitsiou et al., 2002; Takabatake et al., 2000). Moreover, hypoxia‐inducible factor‐1 (HIF‐1), and in particular, its subunit α (HIF‐1α), is overexpressed in lung fibrosis even in the early stage of the disease (Tzouvelekis et al., 2007). Stabilization of HIF‐1α during hypoxia has been associated with an overexpression of muscular DNA damage responses‐1, a negative regulator of the mammalian target of rapamycin (mTOR) pathway (Favier et al., 2015). Hypoxia also increases the expression of myostatin, a muscle‐secreted factor that reduces muscle mass by inhibiting the Akt–mTOR pathway (Amirouche et al., 2009; Hayot et al., 2011). Therefore, chronic hypoxia mostly contributes to muscle wasting by downregulating muscle protein synthesis (Favier et al., 2015). The effects of disease‐induced hypoxia in *f*‐ILD and the mechanisms of action on skeletal muscle mass have yet to be explored in this patient population.

### 3.5 Lifestyle

Physical inactivity is often reported as an important factor contributing to exercise limitation and muscle disuse in ILD (Panagiotou et al., 2016). In this sense, some studies reported that patients take fewer steps per day versus healthy age‐matched subjects (Morino et al., 2017; Wallaert et al., 2013), with this difference reaching ≤65% (Wallaert et al., 2013). As a strategy to avoid facing symptoms (e.g., dyspnoea and fatigue) or owing to fear of exercise (Hoffman et al., 2021; Vainshelboim et al., 2016), patients engage in an inactive lifestyle that can lead to a negative, vicious circle of deconditioning which, in turn, prompts muscle atrophy (Spruit et al., 2013). In fact, Nishiyama et al. (2018) revealed longer sedentary times and a lower engagement even in light physical activity in patients with IPF versus healthy control subjects. It is also key to emphasize that muscle disuse per se can trigger several skeletal muscle alterations observed in patients with *f*‐ILD, such as atrophy or weakness (Booth & Gollnick, 1983).

In addition to physical inactivity, it has been reported that nearly one‐third of patients with IPF present with malnutrition (Jouneau et al., 2019). In fact, recent studies have reported that patients with IPF frequently experience dynamic weight loss (Nakatsuka et al., 2018; Perelas et al., 2019), which can be related to a decreased food intake. Beyond its association with mortality in *f*‐ILD (Comes et al., 2022), harmful effects of malnutrition on skeletal muscles have been described since the 1980s. As shown by Lopes et al. (1982), a lack of nutrient intake decreases energy stores, compromising the contraction and relaxation pattern of the muscle, reducing absolute force and increasing fatigability. Rinaldi et al. (2021) found a moderate association between malnutrition and poorer exercise capacity in *f*‐ILD. However, although muscle loss is a major repercussion of malnutrition in chronic respiratory disease (Gea et al., 2018), a direct insult of poor nutrition status on skeletal muscle function has not yet been explored in *f*‐ILD.

### 3.6 Medications

Corticosteroids are often used in the management of ILD [e.g., in the event of exacerbation (Jang et al., 2021) or for treatment of specific aetiologies (Ejima et al., 2021)]. Long‐term use (≤1 month) has been associated with quadriceps muscle weakness in these patients, with the total amount of medication being inversely related to muscle strength (Hanada et al., 2016). This phenomenon might arise, at least in part, from negative morphological changes, including preferential atrophy of type II muscle fibres (Decramer et al., 1996). Corticosteroids are known to inhibit protein synthesis (e.g., through increased production of myostatin) and increase its degradation [e.g., through the activation of ubiquitin–proteasome and lysosomal systems (Schakman et al., 2009)].

Since the mid‐2010s, antifibrotic drugs [nintedanib (Richeldi et al., 2014) and pirfenidone (King et al., 2014)] have become available for IPF and help to slow down the decline in lung function (Maher & Strek, 2019). Interestingly, antifibrotic therapy in IPF has been associated with a reduction in exercise capacity at 3 months, a decline that was not observed in the treatment‐free group (Iwanami et al., 2022). It has also been shown that patients with IPF on antifibrotic therapy exhibit muscle wasting without lower body mass index versus control subjects (Suzuki et al., 2021). Moreover, a sizeable proportion of patients on antifibrotic therapy experience side‐effects [e.g., diarrhoea, loss of appetite or photosensitivity (Maher & Strek, 2019)] that might promote physical inactivity or malnutrition. These assumptions will require future research exploring a potential link between antifibrotic therapy and muscle deterioration.

### 3.7 Summative evidence

**FIGURE 1:**
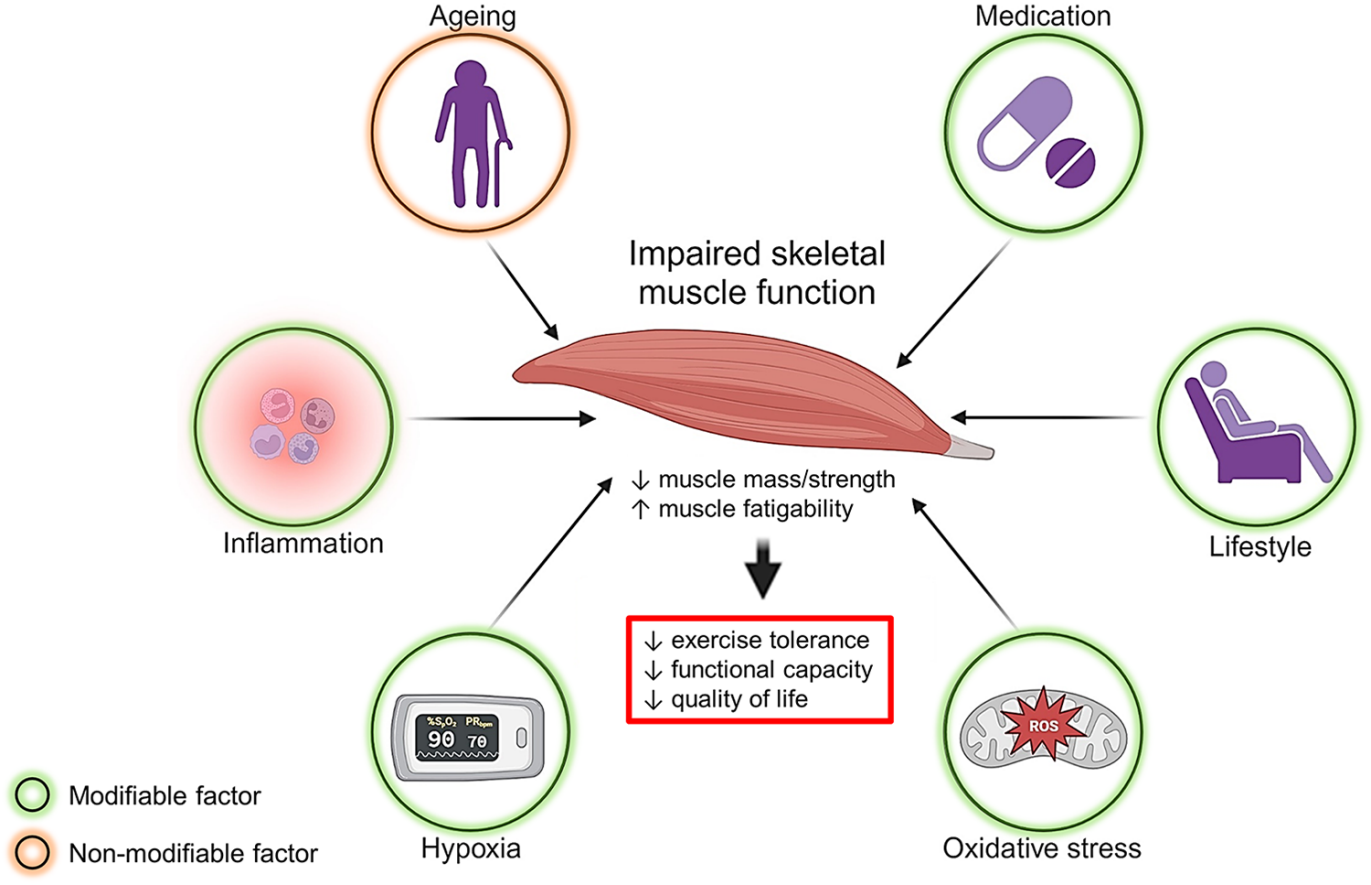
Summary of potential factors that might promote skeletal muscle dysfunction in fibrotic interstitial lung disease and their clinical implications. Although specific evidence on causal relationships may still be missing in this patient population, fibrotic interstitial lung disease is characterized by multiple well‐established factors (depicted as either modifiable or non‐modifiable) promoting muscle dysfunction, including oxidative stress, inflammation, exercise‐induced and chronic hypoxaemia, muscle disuse, malnutrition and corticosteroid intake. Impaired skeletal muscle function has been associated with lower exercise tolerance, functional capacity and quality of life in these patients. See text for further elaboration (figure created with BioRender.com). Abbreviation: ROS, reactive oxygen species.

Although specific evidence on causal relationships is currently lacking, *f*‐ILD is characterized by multiple factors that are known to promote muscle dysfunction. Collectively, these factors (shown as modifiable/non‐modifiable and summarized in Figure 1) in older and physically inactive individuals exposed to repeated courses of corticosteroid use might expose the skeletal muscles of *f*‐ILD patients to a negative milieu.

## 4 WHAT IS THE CURRENT EVIDENCE FOR LOCOMOTOR MUSCLE DYSFUNCTION IN FIBROTIC INTERSTITIAL LUNG DISEASE?

### 4.1 Muscle mass

A relatively small body of evidence suggests that patients with *f*‐ILD experience locomotor muscle atrophy. Specifically, a ∼20% lower cross‐sectional area of the rectus femoris has been reported in advanced *f*‐ILD versus healthy control subjects (Mendes et al., 2015). That study also found lower lower‐limb (triceps surae) but not upper‐limb (biceps brachii) muscle thickness in patients versus control subjects, a pattern of atrophy that is characteristic of muscle disuse [as seen in bedrest studies (Mendes et al., 2015)]. Corroborating evidence of muscle atrophy in *f*‐ILD has been seen using dual‐energy X‐ray absorptiometry; appendicular muscle mass (comprising upper and lower limbs and adjusted for height) was found to be ∼10% lower in a sample of 115 patients (Guler et al., 2019) versus reference values for older individuals (Kelly et al., 2009).

A moderate association was observed between rectus femoris cross‐sectional area and knee‐extensor muscle strength, suggesting that atrophy contributes, at least in part, to muscle weakness in *f*‐ILD (Mendes et al., 2015). In addition, lower appendicular muscle mass was associated with worse pulmonary physiology, as indicated by forced vital capacity or lung diffusing capacity (Guler et al., 2019). Importantly, lower appendicular muscle mass was also correlated with physical performance (quadriceps strength and 6‐min walk distance) and key patient‐reported outcomes (dyspnoea and quality of life) in 27 patients with IPF (Ebihara et al., 2021). Of note, the three highlighted studies did not include any patients with secondary *f*‐ILD (to a systemic disease), with a major focus on idiopathic interstitial pneumonias. Although evidence is currently lacking for the quadriceps, skeletal muscle atrophy (derived from chest CT scans for muscles around the rib cage, e.g., pectoralis) has recently been recognized as a risk factor for mortality in ILD (Molgat‐Seon et al., 2021; Moon et al., 2019; Nakano et al., 2020).

### 4.2 Muscle strength

Although there are limited data available on limb muscle weakness in *f*‐ILD, studies with small sample sizes (typically 20–40 patients) reported lower volitional quadriceps muscle strength versus healthy control subjects or reference values (de Paula et al., 2020; Garcia et al., 2024; Mendes et al., 2015; Nishiyama et al., 2005; Pietro et al., 2017; Watanabe et al., 2013; Zamboti et al., 2021). Collectively, the reduction in quadriceps maximal voluntary contraction approximates 20%–35% in these studies comprising exclusively or a vast majority of idiopathic interstitial pneumonias. One study used magnetic stimulation of the femoral nerve to assess non‐volitional quadriceps muscle strength in a sample of 25 patients with fibrotic idiopathic interstitial pneumonia; its results align with the above‐mentioned research, indicating a 20% reduction in the elicited quadriceps twitch force among patients versus control subjects (Mendoza et al., 2014). Mendes et al. (2015) expanded the examination of locomotor muscle function in *f*‐ILD, exploring plantar‐ and dorsiflexors that are key muscles for walking and balance. Their findings indicated that plantar‐flexors displayed the most important decline in strength (−30% vs. −10% for dorsiflexors) in patients versus control subjects, a pattern that is again consistent with muscle disuse. Muscle weakness has also been reported in secondary ILD, with sarcoidosis being the most‐studied systemic disease. In fact, patients both in early radiographic stages of sarcoidosis (Spruit et al., 2005) and with more advanced lung involvement [∼40% patients with stage III–IV sarcoidosis (Marcellis et al., 2011)] had lower quadriceps muscle strength versus healthy control subjects. This might stem from the fact that disabling symptoms, such as fatigue (reported in ≤80% of participants), reduce daily physical activity, thus promoting physical deconditioning (including muscle weakness) in these patients (Marcellis et al., 2013). This also highlights that findings on muscle impairment and their respective causes might not be extrapolated from one ILD aetiology to another (Panagiotou et al., 2016).

Locomotor muscle weakness is suggested to have key clinical implications for patients with *f*‐ILD. Hence, quadriceps muscle strength was related to or identified as an independent predictor of exercise tolerance (peak O_2_ uptake or work rate) in patients with IPF or sarcoidosis (Nishiyama et al., 2005; Spruit et al., 2005). Impaired functional capacity, typically assessed by but not restricted to the 6‐min walk test, might be another consequence of locomotor muscle weakness (Marcellis et al., 2011; Mendes et al., 2015; Spruit et al., 2005; Watanabe et al., 2013); for instance, Zamboti et al. (2021) showed that patients with ILD (40 participants, of whom 28 had idiopathic interstitial pneumonia) performed more poorly in the timed up‐and‐go test and the sit‐to‐stand tests (30 s, 1 min and five repetitions) versus control subjects, with low quadriceps strength being associated with reduced performance in all these outcomes in patients. As a result of muscle weakness, exercise limitation and related symptoms, impaired quality of life has been suggested in sarcoidosis (Marcellis et al., 2013) and, more recently, in IPF (Ebihara et al., 2021).

### 4.3 Muscle endurance and fatigability

Although it has received less attention, impaired quadriceps muscle endurance has been reported in both mild and severe (i.e. lung transplant candidates requiring supplemental O_2_) ILD. This was evidenced by a ∼20%–25% and ∼45% lower total work performed during an incremental, single‐joint exercise to task failure versus healthy control subjects, respectively (Wickerson et al., 2020). Likewise, Mendoza et al. (2014) used repetitive magnetic stimulation of the femoral nerve to elicit a non‐volitional quadriceps contraction equal to 20% of the maximum voluntary force; the decay in force after 5 min of stimulation indicated muscle endurance. Their findings revealed that quadriceps endurance was lower in patients with fibrotic idiopathic interstitial pneumonia, because their time to decay to 70% of the baseline force was considerably shorter versus healthy control subjects. Interestingly, this result was not related to hypoxia, disease duration or corticosteroid use.

Supporting evidence for increased skeletal muscle fatigability in *f*‐ILD is also available for whole‐body exercise. Specifically, severe exercise‐related hypoxaemia was associated with a **∼**3‐fold blunted increase in quadriceps muscle oxyhaemoglobin throughout constant work‐rate cycling, leading to a ∼50% greater postexercise reduction in quadriceps twitch force by magnetic nerve stimulation in patients versus control subjects (Marillier, Bernard, Verges et al., 2021b). Moreover, increased skeletal muscle fatigability was linked to greater leg muscle symptoms (Marillier, Bernard, Verges et al., 2021b) and a heightened sense of perceived fatigability (Marillier, Gruet, Bernard, Verges et al., 2023) during exercise in *f*‐ILD. Owing to the extreme severity of exercise‐related hypoxaemia in *f*‐ILD, it is conceivable that exaggerated muscle fatigability arises not only from ‘peripheral’ factors [i.e., intrinsic to the muscle (Marillier, Bernard, Verges et al., 2021a)]. Given that hypoxaemia compromises cerebral oxygenation in a dose‐dependant fashion in *f*‐ILD (Marillier, Bernard, Verges et al., 2021a), it remains unclear whether ‘central’ fatigue (i.e., a reduction in the central drive to the skeletal muscles) might also contribute to poorer exercise tolerance in these patients (Marillier, Gruet et al., 2021). In this context, reversing brain hypoxia with supplemental O_2_ on exertion has consistently been associated with better exercise tolerance in *f*‐ILD (Dipla et al., 2021; Marillier, Gruet, Bernard, Champigneulle et al., 2023).

### 4.4 Summative evidence

The pathophysiology of locomotor muscle weakness in ILD remains elusive, because there have been no histological or biological studies to provide direct insights into the underlying causes. Yet, available evidence from accumulating studies with small sample sizes show that quadriceps muscle weakness impacts important outcomes, such as functional capacity and quality of life (Figure 1). A few recent studies have paid attention to locomotor muscle mass and endurance/fatigability, suggesting that those aspects are also impaired in patients with ILD (Table 1). Of note, physical frailty seems to play a mediating role in muscle atrophy and worse muscle function in this population (Tremblay Labrecque et al., 2022). Skeletal muscle atrophy might have a prognostic role, but this has not been shown specifically for the locomotor muscles.

**TABLE 1:** Outline of the main studies assessing peripheral muscle function and the effect of rehabilitative exercise training in patients with fibrotic interstitial lung disease.

Author, year of publication	Study sample	Study design and muscle function outcomes	Main results	Interpretation of results
Skeletal muscle mass				
Mendes et al., 2015	26 patients with advanced ILD (FVC = 49% ± 13% predicted, DL_CO _= 51% ± 20% predicted) 12 healthy controls	Observational, case–control study Rectus femoris CSA, triceps surae and biceps brachii LT (B‐mode ultrasound imaging) Isometric knee‐extensor, plantar‐/dorsi‐flexor and elbow‐flexor MVC	↓ Rectus femoris CSA (7.6 ± 2.1 vs. 9.4 ± 2.4 cm^2^) and triceps surae LT (2.8 ± 0.6 vs. 3.4 ± 0.7 cm) in patients vs. control subjects ↓ Knee‐extensor (119 ± 35 vs. 147 ± 39 Nm) and plantar‐flexor (37 ± 19 vs. 50 ± 15 Nm) MVC in patients versus control subjects Moderate association between elbow‐flexor MVC and biceps LT (*r* = 0.71) and between knee‐extensor MVC and rectus femoris CSA (*r* = 0.63) in ILD	Patients with advanced ILD present with lower‐limb skeletal muscle atrophy and weakness Lower‐limb but not upper‐limb skeletal muscle dysfunction is consistent with muscle disuse
Guler et al., 2019	115 patients with *f*‐ILD [40 with IPF, FVC = 77% ± 13% predicted and DL_CO _= 52% ± 16% predicted in ♂ (*n* = 71), FVC = 72% ± 22% predicted and DL_CO _= 49% ± 17% predicted in ♀ (*n* = 44)]	Observational, single‐group study Skeletal muscle index and upper‐ and lower‐limb muscle mass (dual‐energy X‐ray absorptiometry) Handgrip strength MVC	Moderate/strong association between skeletal muscle index, upper‐ and lower‐limb muscle mass (*r* = 0.67, 0.78 and 0.76, respectively) with handgrip strength Skeletal muscle index and handgrip strength associated with measures of ILD severity (e.g., DL_CO_, % predicted) when adjusted for age, sex and weight	The severity of ILD is associated with lower skeletal muscle mass and strength (and physical performance)
Skeletal muscle strength				
Nishiyama et al., 2005	41 patients with IPF (VC = 77% ± 17% predicted, DL_CO _= 59% ± 20% predicted)	Observational, single‐group study Isokinetic quadriceps MVC ${\dot{V}}_{O_{2}\mathrm{peak}}$ (incremental CPET on a bicycle ergometer)	Quadriceps MVC = 65% ± 15% predicted Quadriceps MVC moderately related to ${\dot{V}}_{O_{2}\mathrm{peak}}$ (*r* = 0.62) and, with VC, independent predictor of ${\dot{V}}_{O_{2}\mathrm{peak}}$	Quadriceps muscle strength is impaired and is a predictor of exercise capacity in IPF
Marcellis et al., 2011	124 patients with sarcoidosis (46 stage III–IV, FVC = 98% ± 21% predicted, DL_CO _= 76% ± 18% predicted) 62 healthy control subjects	Observational, case–control study Isokinetic quadriceps and hamstrings MVC Isometric handgrip and elbow‐flexor MVC	↓ Quadriceps (81 ± 36 vs. 101 ± 301 Nm), hamstrings (62 ± 7 vs. 75 ± 23 Nm) and elbow‐flexor (220 ± 72 vs. 243 ± 72 Nm) MVC in patients versus control subjects ↓ FVC and DL_CO_, fat‐free mass, 6‐min walk distance, and QoL in patients with ↓ upper‐ and/or lower‐limb MVC versus their preserved muscle strength counterparts	Skeletal muscle strength is impaired in sarcoidosis Patients with upper‐ or lower‐limb muscle weakness have lower lung function, exercise capacity and QoL
Skeletal muscle endurance and fatigability				
Mendoza‐Milla et al., 2013	25 patients with *f*‐IIP (15 with IPF, FVC = 79% ±14% predicted, DL_CO _= 40% ± 11% predicted) 33 healthy control subjects	Observational, case–control study Quadriceps twitch force (magnetic stimulation of the femoral nerve) Decay in quadriceps twitch force over 5 min (repetitive magnetic stimulation of the femoral nerve)	↓ Quadriceps twitch force (8.0 ± 2.4 vs. 10.1 ± 3.0 kg) in patients versus control subjects ↑ Decay in quadriceps twitch force [e.g., time to decay to 70% of baseline force = 90 (75; 113) vs. 115 (103; 135) s] in patients versus control subjects	Skeletal (quadriceps) muscle strength and endurance are both reduced in patients with *f*‐ILD
Marillier et al., 2021	15 patients with *f*‐ILD (9 with IPF, FVC = 70% ± 19% predicted, DL_CO _= 44% ± 13% predicted) and severe exercise‐related hypoxaemia 15 healthy control subjects	Interventional, case–control study CWRET (60% of peak work rate): ○ to Tlim breathing air in patients ○ to Tlim_air_ breathing O_2_‐enriched air (fractional inspired O_2 _= 0.42 ± 0.07) in patients ○ to patients’ Tlim_air_ breathing air in control subjects (isotime measures) Quadriceps HbO_2_ concentration (near‐infrared spectroscopy) Quadriceps twitch force (magnetic stimulation of the femoral nerve)	**∼**3‐fold blunted increase in muscle HbO_2_ (+1.3 ± 0.3 vs. 4.4 ± 0.4 µmoL) and ∼50% greater postexercise reduction in quadriceps twitch force (−22.2% ±7.8%% vs. −14.5% ± 9.7%) in patients versus control subjects breathing air Supplemental O_2_ associated with a +5.2 ± 0.5 µmoL in muscle HbO_2_ and −13.3% ± 8.5% decrease in quadriceps twitch force in patients, no longer differing from control subjects	Severe exercise‐related hypoxaemia impairs muscle O_2_ delivery and exacerbates muscle susceptibility to fatigue in ILD Supplemental O_2_ offsets impairments in muscle O_2_ delivery and fatigability in these patients
Rehabilitative exercise training				
Kozu et al., 2011	45 patients with IPF (FVC = 69% ± 16% predicted, DL_CO _= 39% ± 20% predicted) 45 patients with COPD (FVC = 81% ± 23% predicted, DL_CO _= 59% ± 4% predicted) Patients matched for dyspnoea (MRC grade)	Interventional, comparative study Pulmonary rehabilitation: 8 weeks, 2 sessions/week, endurance and resistance training performed Isometric quadriceps MVC Assessed at baseline, immediately after and 6 months post‐programme	Immediately post‐programme: ○ IPF: ∼ ↑ 10% quadriceps MVC ○ COPD: ∼ ↑ 25% quadriceps MVC Blunted ↑ in quadriceps MVC (−0.2 ± 1.0 vs. 3.3 ± 3.5 kg) in patients on oral corticosteroids versus counterparts in IPF Changes in quadriceps MVC and 6‐min walk distance moderately associated (*r* = 0.43) in IPF ↑ in quadriceps MVC not retained at 6 months in IPF (as opposed to COPD)	Rehabilitative exercise training produces only modest, short‐term increase in quadriceps muscle strength in IPF This increase might have been compromised by daily intake of oral corticosteroids in IPF
Dowman et al., 2017	142 patients with *f*‐ILD (61 with IPF, FVC ∼75% predicted, DL_CO_ ∼50% predicted)	Interventional, comparative study Patients randomized to either: ○ Rehabilitative exercise training (8 weeks, 2 sessions/week, endurance and resistance training performed) ○ Usual care Knee‐extensor and elbow‐flexor MVC (hand‐held dynamometry) Assessed at baseline, immediately after and 6 months post‐programme	Mean difference (95% CI) in muscle strength (in kilograms) between exercise and usual care groups did not reach statistical significance. Immediately post‐programme: ○ Knee‐extensor MVC: 1.3 (−0.1; 2.6) ○ Elbow‐flexor MVC: 0.3 (−0.7; 2.6) 6 months post‐program: ○ Knee‐extensor MVC: 0.8 (−0.7; 2.2) ○ Elbow‐flexor MVC: −0.2 (−1.2; 2.2) ↑ in 6‐min walk distance and quality of life in the exercise training group immediately post‐programme; benefits declined at 6 months post‐programme	Rehabilitative exercise training is effective in patients with *f*‐ILD across the range of different aetiologies, but not for skeletal muscle strength specifically Benefits declined at 6 months post‐programme in the whole sample, which differs according to disease aetiology or severity
Perez‐Bogerd et al., 2018	60 patients with *f*‐ILD (14 with IPF, SVC ∼75% predicted, DL_CO_ ∼40%–45% predicted)	Interventional, comparative study Patients randomized to either: ○ Pulmonary rehabilitation (6 months, 2–3 sessions/week, endurance and resistance training performed) ○ Usual care Isometric quadriceps and handgrip MVC Assessed at baseline and at 3, 6 and 12 months (6 months post‐programme)	Mean difference (95% CI) in muscle strength (as a percentage of predicted) between exercise and usual care groups reach statistical significance except for handgrip MVC at 12 months Isometric quadriceps MVC: ○ At 3 months: 10 (2; 18) ○ At 6 months: 10 (1; 18) ○ At 12 months: 10 (1; 18) Isometric handgrip MVC: ○ At 3 months: 13 (5; 20) ○ At 6 months: 12 (4; 20) ○ At 12 months: 6 (−3; 15)	Rehabilitative exercise training improves skeletal (quadriceps) muscle strength These benefits are maintained at 12 months, i.e., 6 months post‐programme Maintenance might be explained by the long‐lasting (i.e., 6 months) nature of the programme
Keyser et al., 2015	13 patients with *f*‐ILD (3 with IPF, FVC = 53% ± 18% predicted, DL_CO _= 39% ± 15% predicted)	Interventional, single‐group study Rehabilitative exercise training: 10 weeks, 3 sessions/week, endurance (treadmill) training performed Cardiac output (bioimpedance cardiography) and gastrocnemius HHb concentration (near‐infrared spectroscopy) during a treadmill CPET Assessed at baseline and immediately post‐programme	Immediately post‐program at the peak of treadmill CPET: ↑ ${\dot{V}}_{O_{2}\mathrm{peak}}$ with no change in cardiac output and stroke volume ↑ Arteriovenous O_2_ difference (+16%) and muscle HHb concentration (∼+18 vs. +25 a.u.) ↑ Arteriovenous O_2_ difference and muscle HHb concentration moderately associated (*r* = 0.51) after exercise training	Increases in exercise capacity seem to be mediated by enhanced skeletal muscle O_2_ extraction capacity rather than by changes in O_2_ delivery after exercise training in *f*‐ILD

Abbreviations: CI, confidence interval; COPD, chronic obstructive pulmonary disease; CPET, cardiopulmonary exercise testing; CSA, cross‐sectional area; CWRET, constant work rate exercise test; DL_CO_, lung diffusing capacity for carbon monoxide; *f*‐ILD, fibrotic interstitial lung disease; FVC, forced vital capacity; HbO_2_, oxyhaemoglobin; HHb, deoxyhaemoglobin; ILD, interstitial lung disease; IPF, idiopathic pulmonary fibrosis; LT, layer thickness; MVC, maximal voluntary contraction; QoL, quality of life; SVC, slow vital capacity; Tlim, time to intolerance; VC, vital capacity; ${\dot{V}}_{O_{2}\mathrm{peak}}$, peak oxygen uptake.

## 5 CAN REHABILITATIVE EXERCISE TRAINING REVERSE SKELETAL MUSCLE ABNORMALITIES IN FIBROTIC INTERSTITIAL LUNG DISEASE?

### 5.1 Muscle mass and strength

We found three studies that assessed the influence of exercise training/pulmonary rehabilitation on quadriceps muscle strength in *f*‐ILD (including, at least, a substantial fraction of patients with IPF or idiopathic interstitial pneumonias) with rather conflicting results. Each study offered a combined endurance and resistance training programme; however, Dowman et al. (2017) found no effect of the intervention on peripheral muscle strength, whereas studies by Kozu et al. (2011) and Perez‐Bogerd et al. (2018) reported a ∼10% increase in this outcome post‐programme. These investigations all delivered two or three weekly sessions, but the study by Perez‐Bogerd et al. (2018) had a total duration of 6 months, whereas the other studies were limited to 8 weeks. This might explain why the study by Perez‐Bogerd et al. (2018) was the only one to report long‐lasting (i.e., at 6 months) effects on muscle strength post‐intervention. The post‐intervention magnitude of increase in muscle strength does not seem particularly large in *f*‐ILD; a ∼2.5‐fold greater increase was seen in chronic obstructive pulmonary disease (COPD; +25%) after a similar programme [although the intensity of exercise during the endurance training differed among populations (Kozu et al., 2011)]. For the purpose of comparability, the reported increase in COPD coincides with what is typically found after combined endurance and resistance training (∼+30%, as reviewed by De Brandt et al., 2018).

Kozu et al. (2011) were also the only authors reporting a significant, moderate association between changes in quadriceps muscle strength and the functional capacity of patients (as assessed by the 6‐min walk test) in *f*‐ILD, although strength gains might have been compromised by the daily intake of oral corticosteroids. This study, however, enrolled a relatively low number (*n* = 36) of patients with IPF. This probably impedes the generalizability of its results to other aetiologies of *f*‐ILD. None of these studies reported whether changes in quadriceps muscle strength relates to important patient‐centred outcomes, such as health‐related quality of life. Interestingly, a preliminary randomized controlled trial of 18 patients with late‐stage (III and IV) sarcoidosis reported that a 12‐week exercise programme (24 sessions) improved leg muscle strength by a median of 10 kg [∼18% (Naz et al., 2018)]. These findings were concomitant to improvements in health‐related quality of life and anxiety but, again, whether changes in leg muscle strength are related to these outcomes was not examined.

Although muscle atrophy (at least in the lower limb) has been reported previously in advanced *f*‐ILD (Mendes et al., 2015), no study to date has evaluated whether exercise training might reverse this impairment in this patient population. Of note, skeletal muscle size (rectus femoris cross‐sectional area) and strength (knee extensors) were relatively well associated (Mendes et al., 2015). Owing to the fact that exercise training might improve muscle strength (although gains might be minimal, see above), it is likely that these changes might be accompanied by increases in muscle mass in *f*‐ILD (Holland, 2015). If one modality would need to be chosen to target muscle mass, resistance should be prioritized over endurance training [or both combined (Menon, Houchen, Harrison et al., 2012, 2012)].

### 5.2 Muscle endurance and fatigability

Data regarding isolated muscle endurance after exercise training are currently lacking in *f*‐ILD (no study reporting this specific outcome was identified). It is, however, conceivable that this intervention might improve muscle endurance in this population. A few studies have shown an increase of 50%–60% after 4–8 weeks of aerobic exercise performed at ∼40%–65% (Vivodtzev et al., 2010) and 50%–80% (Covey et al., 2014) or peak work rate in COPD. On the contrary, lower or limited progression of exercise intensity over the course of endurance training might be expected in *f*‐ILD (Holland et al., 2015); exercise intensity was ∼20% lower at the end of an 8‐week programme versus COPD, owing to distressing dyspnoea, cough or profound hypoxaemia (Kozu et al., 2011). The magnitude of improvement in muscle endurance, if present, might thus be hampered after training in *f*‐ILD.

One study specifically investigated muscle oxygenation parameters (by near‐infrared spectroscopy) during exercise before and after rehabilitative exercise training in *f*‐ILD (Keyser et al., 2015). The authors reported an increase in exercise capacity (i.e., peak O_2_ uptake) in the absence of changes in cardiac output after the intervention during an incremental treadmill exercise test. Arteriovenous O_2_ difference (calculated as peak O_2_ uptake divided by cardiac output) and the change in gastrocnemius deoxyhaemoglobin concentration (reflecting muscle O_2_ extraction capacity) were, however, significantly improved. It was concluded that increases in exercise capacity were mediated by enhanced skeletal muscle O_2_ extraction capacity rather than changes in O_2_ delivery after exercise training in *f*‐ILD. It remains untested whether this mechanism would allow skeletal muscle fatigue to develop more after exercise in *f*‐ILD [using non‐volitional techniques, for instance (Marillier et al., 2021)]. In fact, the ability to induce skeletal muscle fatigue during an exercise training session has been linked to a larger improvement in functional capacity and quality of life after the intervention was completed (Burtin et al., 2012).

### 5.3 Summative evidence

**FIGURE 2:**
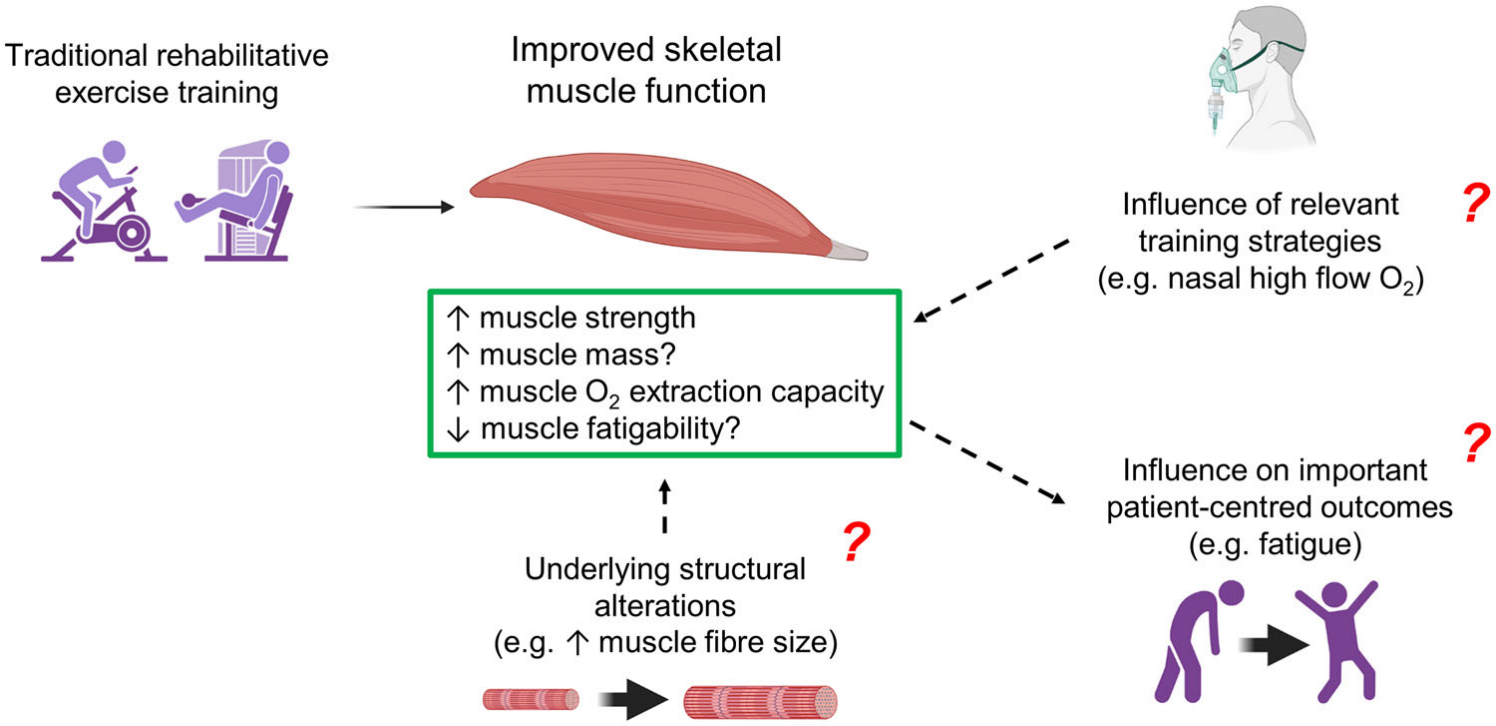
Summary of currently available evidence about the effects of rehabilitative exercise training on skeletal muscle function and potential research avenues in fibrotic interstitial lung disease. Briefly, the intervention improves muscle strength, although the magnitude of improvement does not seem particularly large; it remains unknown whether it accompanies positive changes in muscle mass. Improved muscle O_2_ extraction capacity has been suggested after endurance training. The underlying structural alterations leading to improved muscle function and the influence of such improvement on key patient‐centred outcomes have not yet been investigated in fibrotic interstitial lung disease. This also holds true for alternative training strategies targeting skeletal muscles. See text for further elaboration (figure created with BioRender.com).

Locomotor muscle dysfunction is associated with poor exercise tolerance (Nishiyama et al., 2005), functional capacity (Mendes et al., 2015; Watanabe et al., 2013) and quality of life (Spruit et al., 2005) in *f*‐ILD. As exposed, muscle abnormalities have received little attention in this patient population; only a few studies reported minimal, inconsistent improvements in skeletal muscle strength after rehabilitative exercise training. This might stem from the fact that patients cannot tolerate sufficiently high training intensities (owing to unbearable symptoms or severe hypoxaemia) to derive large benefits. High drop‐out rates (∼30% in these specific studies) owing to, for instance, exacerbations or poor attendance, might also impede the efficacy of the intervention. Although improvements in skeletal muscle mass and endurance can be expected after training in *f*‐ILD, it remains unknown whether they would be accompanied secondary to changes in muscle micro‐ and/or macro‐structure (muscle fibre typing or capillarization, Figure 2).

## 6 PERSPECTIVES: HOW CAN THE MANAGEMENT OF LOCOMOTOR MUSCLE DYSFUNCTION IN FIBROTIC INTERSTITIAL LUNG DISEASE BE OPTIMIZED?

### 6.1 Offering relevant alternative training strategies

#### 6.1.1 Interval‐based endurance exercise training

Owing to excessive dyspnoea or exertional desaturation, some people with *f*‐ILD might not be able to tolerate moderate‐intensity continuous training, traditionally offered during exercise training (Dowman, May et al., 2021). The net result might be a reduced training stimulus, thus limiting programme benefits. Interval training [relatively short, high‐intensity bouts of exercise interspaced by periods of recovery (Marillier et al., 2022)] might be a promising and efficacious alternative to continuous training in *f*‐ILD. Two ‘proof‐of‐concept’ studies (nine participants each) reported that a single session of interval training was associated with less desaturation (2%–3%) and a greater preference in 95% of patients in comparison to continuous training, without placing excessive demands on the cardiorespiratory system in *f*‐ILD (Dowman, May et al., 2021; Wickerson et al., 2021). Thus, interval exercise delivers a high‐intensity training stimulus to peripheral muscles with no harmful influence on perceptual responses [leg discomfort, in particular (Dowman, May et al., 2021; Wickerson et al., 2021)]. Future studies might examine whether this modality offers greater improvements (in muscle‐centred outcomes) following a full programme, in comparison to continuous training in *f*‐ILD. For instance, interval training has been shown to be particularly effective in improving muscle fibre type in the vastus lateralis across all COPD stages (Vogiatzis et al., 2007, 2005, 2011). Importantly, the acute affective response to exercise can predict long‐term participation in physical activity (Williams et al., 2008), which might favour the implementation of interval training in *f*‐ILD.

#### 6.1.2 Nasal high‐flow O_2_ therapy

Heated and humidified nasal high‐flow O_2_ therapy has recently become a promising method in patients with respiratory disorders, typically allowing O_2_ to be delivered at flow rates of 30–60 L/min and an inspired fraction of ∼0.5 on exertion (Al Chikhanie et al., 2021; Spoletini et al., 2015). This option is particularly relevant for patients with *f*‐ILD, who characteristically require O_2_ flows at which standard O_2_ therapy with a nasal cannula are poorly tolerated (Lee et al., 2016). Beyond improving oxygenation, nasal high‐flow O_2_ therapy offers additional physiological benefits, including reduced work of breathing (Vargas et al., 2015), improved breathing pattern (Braunlich et al., 2013) and clearance of dead space (Moller et al., 2015). Two pilot studies (35 patients overall) showed that nasal high‐flow O_2_ prolonged the endurance time to symptom limitation versus standard O_2_ therapy in *f*‐ILD (Al Chikhanie et al., 2021; Badenes‐Bonet et al., 2021). This was found with greater systemic and skeletal muscle oxygenation (Al Chikhanie et al., 2021; Badenes‐Bonet et al., 2021) and less symptoms of leg fatigue at isotime (Al Chikhanie et al., 2021). These observations are of clinical relevance, because enhancing muscle oxygenation with O_2_ supplementation led to improved quadriceps muscle fatigability during exercise in *f*‐ILD (Marillier, Bernard, Verges et al., 2021b). Taken together, it is conceivable that nasal high‐flow O_2_ might facilitate exercise training in this patient population, acting as an ergogenic aid to the skeletal muscles (Figure 2).

#### 6.1.3 Water‐based endurance exercise training

It has been proposed that water‐based training might prove particularly useful in patients with comorbidities (e.g., obesity or musculoskeletal conditions) to ease participation (McNamara et al., 2013), offering similar upper‐ and lower‐body strength gains to a land‐based programme (Felcar et al., 2018). Obesity is found in ∼35% of patients with *f*‐ILD (Comes et al., 2022), can worsen lung restriction per se (Alqurashi et al., 2024; Marillier et al., 2020) and is usually associated with low levels of physical activity (Tudor‐Locke et al., 2010). Some aetiologies of *f*‐ILD (e.g., sarcoidosis or rheumatoid arthritis) can also be associated with musculoskeletal limitations (Nessrine et al., 2014; Sparks, 2019). In this context, water‐based training might be a relevant substitute for land‐based training in *f*‐ILD. However, it might be feasible only in patients with mild *f*‐ILD, because O_2_ therapy cannot be used [i.e., portable devices, such as liquid O_2_ canisters, for advanced patients requiring high O_2_ flow rates (Jacobs et al., 2020)]. Alternatively, lessened hypoxaemia can be anticipated with weight‐bearing activities (Palange et al., 2000), which might favour the implementation of water‐based exercise training in *f*‐ILD.

#### 6.1.4 Elastic band resistance training

Interest in low‐load, high‐repetition elastic band resistance training has recently emerged in COPD to overcome the limited availability of specific equipment (Nyberg et al., 2015). After 8 weeks, upper‐ and lower‐limb muscle strength and endurance increased by ∼10% and 15%, respectively, alongside gains in functional capacity. Of note, partitioning the exercise (i.e., using a single limb vs. two) during elastic band resistance training allowed greater total work to be performed (∼25%) leading to greater quadriceps fatigue (∼30%), with similar or lower levels of exertional symptoms (Nyberg et al., 2016). Although recommended as part of the early management of progressive *f*‐ILD (Raghu et al., 2022), it has been shown that only ∼20% of patients with IPF were referred to pulmonary rehabilitation (de Andrade et al., 2021) and 5%–10% eventually underwent it (Behr et al., 2015; Fernandez‐Fabrellas et al., 2019). This arises, at least in part, from lack of access to a local pulmonary rehabilitation centre (de Andrade et al., 2021; Hoffman et al., 2021). Home‐based rehabilitation is currently in its infancy in *f*‐ILD (Cerdan‐de‐Las‐Heras et al., 2021). In this context, elastic band resistance training might prove useful both in extending the access to rehabilitation to the home and in being effective for improving skeletal muscle function in this patient population.

### 6.2 Focusing on other relevant muscle properties and functional testing modalities

In addition to strength and endurance, muscle power (i.e., the ability to produce energy in a short period of time) is an important functional muscle property; it can be assessed either via dynamic ergometer measurements (e.g., isokinetic protocols) or by functional tests [e.g., five‐repetition sit‐to‐stand test (Bui et al., 2017)]. In fact, it has been shown that, among functional muscle properties, muscle power was the best predictor of patients’ 6‐min walk distance and 1‐min sit‐to‐stand repetitions, suggesting that it might be more relevant to functional exercise capacity than muscle strength and endurance (Gephine et al., 2021). Importantly, when an intervention is chosen to enhance muscle strength and power (such as resistance exercise training), a sit‐to‐stand test would be more relevant than the ‘traditional’ 6‐min walk test to assess its influence on the functional capacity of patients, considering the requirements of both tests (De Brandt et al., 2022). Of note, the validity of the 1‐min sit‐to‐stand test specifically to assess the functional exercise capacity of patients in ILD was documented recently (Tremblay Labrecque et al., 2020). In this context, although patients with *f*‐ILD typically perform poorly versus control subjects in terms of both muscle power (five‐repetition sit‐to‐stand test) and functional exercise capacity [1‐min sit‐to‐stand test (Tremblay Labrecque et al., 2022; Zamboti et al., 2021)], frailty seems, again, to have a mediating role in worse outcomes in patients (Tremblay Labrecque et al., 2022). Functional testing in *f*‐ILD might also be extended to the stair‐climb power test, because its properties are more closely associated with mobility limitations than muscle strength (Bean et al., 2003). Altogether, future research exploring the effect of rehabilitative exercise training on locomotor muscle function in *f*‐ILD should consider muscle power as key muscle property and how its improvement translates into enhanced functional exercise capacity with relevant functional testing modalities.

### 6.3 Characterizing underlying micro‐ and macro‐structural muscle abnormalities

Muscle disuse plays an indisputable role in muscle dysfunction in *f*‐ILD (Panagiotou et al., 2016). However, specific hallmarks of *f*‐ILD (e.g., frequent hypoxaemia or repeated courses of corticosteroid use; for details, see section 1) might additionally conspire to aggravate muscle structural and functional abnormalities. Given that recent systematic reviews show that patients are markedly inactive (Iwakura et al., 2023; Rocha et al., 2022), with drastically reduced daily levels of physical activity versus healthy individuals (Wallaert et al., 2013), teasing out the respective contribution of muscle disuse from factors specific to *f*‐ILD on these muscle‐centred outcomes will require enrolling well‐matched control subjects to address this concern. Nonetheless, robust evidence suggests that beneficial changes in muscle characteristics can be seen after endurance and resistance exercise training (for review, see Booth et al., 2015; Marillier et al., 2019; McGlory et al., 2017). Muscle biopsy‐based studies would help to provide a clearer picture on: (1) whether expected micro‐ and macro‐structural muscle abnormalities could be explained entirely by muscle disuse attributable to physical inactivity or whether factors inherent to *f*‐ILD might also be involved; and (2) whether endurance and resistance exercise training could have beneficial (and probably synergic) effects on muscle structure and their related functional properties in *f*‐ILD. It is conceivable that no biopsy‐based studies are available so far owing to: (1) the recent interest in and emerging evidence for muscle dysfunction and exercise training in *f*‐ILD (Curtis & Hopkinson, 2017); and (2) the poor prognosis typically associated with the most devastating forms of *f*‐ILD, namely IPF or, more broadly, progressive pulmonary fibrosis (Martinez et al., 2017; Wijsenbeek et al., 2019), which might ethically preclude offering the procedure to these patients. This might be counter‐balanced by: (1) the call for rehabilitative exercise training as part of the holistic management of these patients (Oliveira et al., 2023), which has the potential to alter muscle micro‐ and micro‐structure (see above); and (2) crucial pharmacological advances (nerandomilast) slowing down disease progression beyond what previous antifibrotic therapy usually offered in both IPF and progressive pulmonary fibrosis (Maher et al., 2025; Richeldi et al., 2025), which might suggest that these studies will be conducted in the near future.

### 6.4 Focusing more on patient‐centred outcomes

Patient‐centred outcome research in ILD was recently defined as a ‘collection of reliable and valid endpoints for research that represent what matters most to individual patients in their day‐to‐day lives’ (Aronson et al., 2021). Key patient‐centred outcomes that are reasonably susceptible to alteration by rehabilitative exercise training and muscle‐related improvements in this group of diseases include health‐related quality of life, symptoms, functional status and psychological and emotional well‐being (Aronson et al., 2021). Of note, the statement emphasized that future studies should explore fatigue as an endpoint, using validated outcome measures in these patients. In this context, the Fatigue Severity Scale has shown appropriate validity and reliability in a large cohort of 1881 patients (about two‐thirds with IPF) from the Pulmonary Fibrosis Foundation Patient Registry (Aronson et al., 2023). It has to be acknowledged that fatigue has received attention in sarcoidosis [since being reported in ≤80% of patients (Marcellis et al., 2013)], including concomitant improvement in fatigue and quadriceps muscle strength after exercise training in a small group of patients in early radiographic stages (Marcellis et al., 2015). However, it is also key to emphasize that symptoms of fatigue are extremely prevalent and of clinical relevance in *f*‐ILD, because ∼70% of patients seek medical advice owing to fatigue, regardless of the disease aetiology (Guenther et al., 2018). Exploring whether improvement of muscle function with rehabilitative exercise training might translate into lessened symptoms of fatigue (or other key patient‐centred outcomes) is crucial in upcoming studies of *f*‐ILD (Figure 2).

## 7 CONCLUSION

This narrative review found robust evidence in favour of locomotor muscle dysfunction in *f*‐ILD, with implications for exercise limitation. However, its pathophysiology remains elusive, because no biopsy‐based studies have been conducted to provide insights into underlying causes (i.e., muscle disuse and/or mechanisms specific to *f*‐ILD). Collectively, this review highlights that the ability of rehabilitative exercise training to reverse muscle abnormalities has received little attention in this population (at least in idiopathic interstitial pneumonias); only a few studies have reported minimal, inconsistent improvements in skeletal muscle strength. This might stem from the fact that patients cannot tolerate sufficiently high training intensities, owing to unbearable symptoms or severe hypoxaemia, to derive substantial benefits. Future research should, therefore, focus on optimizing the effectiveness of rehabilitative exercise training as related to locomotor muscle function and how this might translate into improvements in key patient‐centred outcomes. Lastly, although we aimed to be as exhaustive as possible, we acknowledge the inherent limitations of the present manuscript written as a narrative review; these include a restricted search methodology in comparison to a systematic approach, no formal assessment of the quality of studies, and thus, no method intended to minimize bias. A systematic review as related to locomotor muscle function and rehabilitative exercise training in *f*‐ILD might prove useful to address such limitations once substantial literature becomes available.

## AUTHOR CONTRIBUTIONS

Conceptualization: M.M.; Acquisition, analysis, or interpretation of data: S.T., J.A.N., A.‐C.B., M.C., M.G., F.H., S.V. and M.M.; Drafting the work or revising it critically for important intellectual content—original draft: S.T. and M.M.; revised draft: J.A.N., A.‐C.B., M.C., M.G., F.H. and S.V. All authors approved the final version of the manuscript and agree to be accountable for all aspects of the work in ensuring that questions related to the accuracy or integrity of any part of the work are appropriately investigated and resolved. All persons designated as authors qualify for authorship, and all those who qualify for authorship are listed.

## CONFLICT OF INTEREST

The authors declare that this research was conducted in the absence of any commercial or financial relationships that could be construed as a potential conflict of interest.
